# Effects of Germination and Alcalase Hydrolysis on the Compositional and Bioactive Properties of Soybean Flour and Soy Protein Isolate

**DOI:** 10.3390/foods15213988

**Published:** 2026-11-06

**Authors:** Jiranan Ratseewo, Nidthaya Seephua, Sirithon Siriamornpun

**Affiliations:** 1 Division of Food Innovation and Technology, Faculty of Liberal Arts and Science, Sisaket Rajabhat University, Sisaket 33000, Thailand; jiranan.r@gmail.com; 2 Department of Food Technology and Nutrition, Faculty of Technology, Mahasarakham University, Maha Sarakham 44150, Thailand; nidthaya.s@msu.ac.th; 3 Research Unit of Thai Food Innovation (TFI), Mahasarakham University, Maha Sarakham 44150, Thailand

**Keywords:** enzymatic processing, proteolysis, phenolic compounds, antioxidant activity, soy protein isolate

## Abstract

Germination and enzymatic hydrolysis can alter the composition and bioactive properties of soybean ingredients. This study compared soybean flour and soy protein isolate (SPI) processing routes involving germination (0, 24, or 72 h) and Alcalase hydrolysis (40 or 60 °C). Water-extractable protein, lipid content, phenolic and flavonoid contents, antioxidant activity (DPPH and FRAP), GABA, and free amino acid profiles were determined using spectrophotometric and chromatographic methods. The 72 h-germinated SPI hydrolyzed at 60 °C (SG72IE60) showed the highest Bradford-measured water-extractable protein value (23.04 mg/g db). Among flour-based treatments, SG72E60 had the highest apparent total phenolic content (75.03 mg GAE/100 g db), flavonoid content (55.74 mg RE/100 g db), DPPH activity (55.73 mg TE/g db), and FRAP value (55.91 mg FeSO_4_/g db). Germination for 72 h increased γ-aminobutyric acid (GABA) content from 0.25 to 2.92 mg/g db. The highest total concentration of quantified free essential amino acids was observed in SG24E60 (1032.77 µg/g db), with no significant difference from SE60 (1027.11 µg/g db; *p* > 0.05). Leucine was the most abundant essential amino acid in SG24E60 (548.54 µg/g db). In contrast, SE60 exhibited the highest total concentration of quantified free non-essential amino acids (344.78 µg/g db), with tyrosine being the predominant amino acid (167.89 µg/g db). Overall, the results indicate that different processing conditions favored distinct compositional and bioactive characteristics.

## 1. Introduction

Soybean (*Glycine max* L.) is an important plant-based food resource because of its high protein content, favorable amino acid composition, and abundance of bioactive constituents [1,2]. Soybean proteins, particularly the major storage proteins glycinin and β-conglycinin, provide a valuable source of dietary amino acids and can be converted into peptides and free amino acids through proteolytic processing [3]. In addition to proteins, soybeans contain phenolic compounds, isoflavones, and other phytochemicals that contribute to their antioxidant properties [3,4]. Consequently, processing strategies capable of improving the accessibility of soybean proteins and enhancing bioactive components are of considerable interest for the development of value-added and functional food ingredients.

Germination is a biological processing approach that induces extensive biochemical and structural changes in seeds. During germination, endogenous hydrolytic enzymes are activated to mobilize stored macromolecules required for seedling development, resulting in changes in proteins, lipids, carbohydrates, and secondary metabolites [5,6]. In soybean, germination has been associated with changes in phenolic compounds, isoflavones, amino acids, and antioxidant capacity [7,8,9]. Germination is also particularly relevant to the accumulation of γ-aminobutyric acid (GABA), a non-protein amino acid formed primarily through the decarboxylation of glutamate by glutamate decarboxylase as part of the GABA shunt. Recent studies have demonstrated substantial changes in GABA and phenolic metabolism during legume germination [10,11], while Xu et al. [4] demonstrated that GABA accumulation in germinated soybean is closely associated with the regulation of glutamate metabolism and GABA-related enzymes. These metabolic responses indicate that germination duration may be an important determinant of the nutritional and bioactive composition of soybean-derived ingredients.

Enzymatic hydrolysis provides an additional strategy for modifying soybean proteins. Alcalase, a broad-specificity serine endoprotease, has been extensively applied to plant proteins because of its ability to hydrolyze protein structures and generate lower-molecular-weight protein fractions. Alcalase hydrolysis of soybean proteins has been shown to modify protein structure, peptide profiles, physicochemical properties, and biological activities [12,13]. More recent evidence indicates that enzymatic hydrolysis progressively disrupts major soybean storage proteins, including glycinin and β-conglycinin, and promotes the formation of low-molecular-weight peptide fractions [14,15,16]. Such proteolytic changes may increase the water-extractable protein fraction and alter the free amino acid profile of soybean materials. The characteristics of the resulting hydrolysates are strongly dependent on processing conditions, including enzyme type, temperature, pH, substrate characteristics, and hydrolysis duration.

Previous studies have demonstrated that soybean germination and Alcalase hydrolysis can modify protein characteristics, phenolic constituents, and antioxidant-related properties [7,12,17,18]. Although these processing techniques are well established, comparative information on the compositional and bioactive profiles obtained through direct Alcalase hydrolysis of soybean flour and SPI preparation followed by hydrolysis remains limited. These processing routes differ in the composition of the materials subjected to enzymatic hydrolysis. Soybean flour retains proteins and non-protein constituents, whereas SPI preparation involves defatting, alkaline extraction, and isoelectric precipitation, which may influence the recovery of matrix-associated compounds and the characteristics of the resulting protein fraction [19,20]. Comparing these routes under corresponding germination durations and hydrolysis temperatures may therefore provide insight into differences in water-extractable protein, free amino acids, GABA, phenolic and flavonoid contents, and antioxidant-related properties. Such a comparison may also reveal processing-related trade-offs, as conditions associated with higher values for one characteristic may not produce the highest values for others.

Accordingly, this study aimed to compare the compositional and bioactive properties of soybean flour and SPI-derived hydrolysates obtained through different germination durations and Alcalase hydrolysis temperatures. Particular attention was given to the differences between direct hydrolysis of soybean flour and SPI preparation followed by hydrolysis, providing comparative evidence to support the selection of processing routes according to specific compositional and bioactive priorities.

## 2. Materials and Methods

### 2.1. Chemicals and Reagents

Soybean (*Glycine max* (L.) Merr.) seeds harvested in 2025 were purchased in Roi Et Province, Thailand. Alcalase^®^ 2.4 L, a proteolytic enzyme preparation derived from *Bacillus licheniformis*, was supplied by Novozymes A/S (Bagsværd, Denmark). Amino acid standards, Folin–Ciocalteu reagent, 2,4,6-tripyridyl-*s*-triazine (TPTZ), 2,2-diphenyl-1-picrylhydrazyl (DPPH), and bovine serum albumin (BSA) were purchased from Sigma–Aldrich Co. (St. Louis, MO, USA). All other analytical-grade chemicals, HPLC-grade solvents, and reference standards (purity ≥ 90%) were also obtained from Sigma-Aldrich (St. Louis, MO, USA).

### 2.2. Experimental Design and Sample Coding

**Figure 1:**
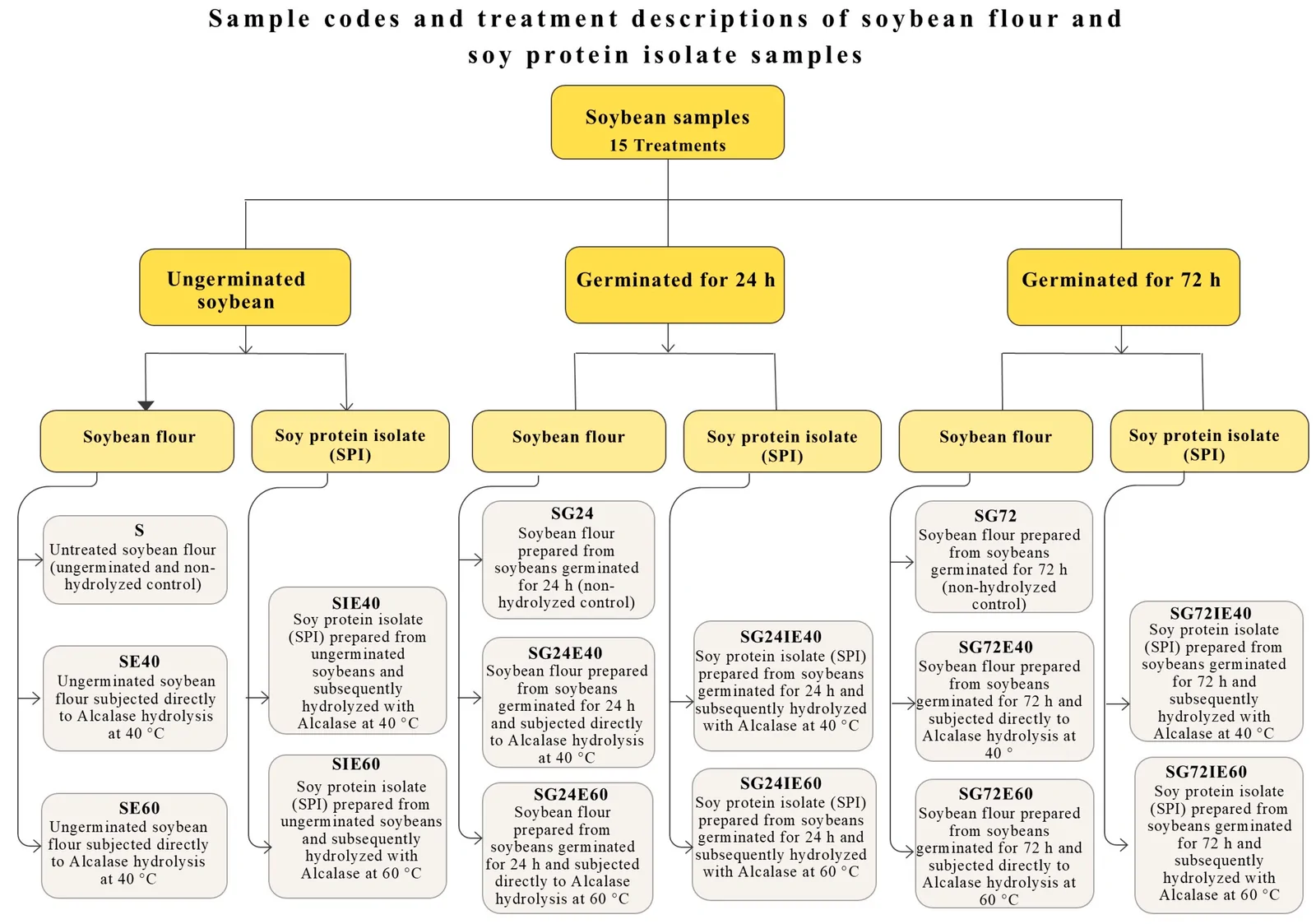
Sample codes and treatment descriptions of soybean flour and soy protein isolate samples.

The experiment was arranged in a completely randomized design (CRD) comprising 15 distinct processing treatments (Figure 1). Each treatment was independently prepared in three separate batches. Each batch represented one independent experimental replicate and was considered one experimental unit, resulting in a total of 45 experimental units (15 treatments × 3 independent batches). Two processing routes were included: the soybean flour route and the soy protein isolate (SPI) route. In the soybean flour route, non-germinated and germinated soybeans were either retained without enzymatic hydrolysis or directly hydrolysed with Alcalase^®^ at 40 or 60 °C. In the SPI route, non-germinated or germinated soybeans were first processed into SPI and subsequently hydrolysed with Alcalase^®^ at 40 or 60 °C. For germinated samples, germination was performed before flour or SPI preparation.

The treatment codes were defined as follows: S, soybean; G24 and G72, germination for 24 and 72 h, respectively; I, preparation of SPI; and E40 and E60, Alcalase hydrolysis at 40 and 60 °C, respectively. Accordingly, the 15 treatments were S, SE40, SE60, SG24, SG24E40, SG24E60, SG72, SG72E40, SG72E60, SIE40, SIE60, SG24IE40, SG24IE60, SG72IE40, and SG72IE60. The position of I before E indicates that SPI preparation preceded enzymatic hydrolysis. The letter I therefore represents SPI preparation and was not considered an independent defatting treatment.

The experimental design comprised 15 distinct processing treatments rather than a complete factorial arrangement. Non-hydrolyzed soybean samples served as reference treatments for the corresponding germination durations, while non-hydrolyzed SPI controls were not included. The study was designed to compare the compositional and bioactive properties of soybean flour and SPI-derived hydrolysates across different germination durations and hydrolysis temperatures. Therefore, differences between the two processing routes were interpreted as overall processing-related differences rather than the independent or interactive effects of SPI preparation and Alcalase hydrolysis.

### 2.3. Sample Preparation

Soybean samples were prepared according to Yang et al. [21] with modifications to the soaking conditions, germination period, and drying method. Soybean seeds were washed twice with clean water and soaked in water at a soybean-to-water ratio of 1:10 (*w*/*v*) for 12 h. The soaked seeds were drained and germinated in the dark at ambient temperature for either 24 or 72 h. During germination, the seeds were rinsed daily with clean water.

The germinated soybeans were dried in a hot-air oven (UFE 600, Memmert Company, Memmert, Germany) at 60 °C until their moisture content reached approximately 10%. Non-germinated and dried germinated soybeans were separately ground and passed through a 60-mesh sieve to obtain homogeneous soybean flour. The resulting powders were packed in aluminium foil pouches and stored at 4 °C until further processing and analysis. Moisture content was determined using the AOAC Official Method [22] and expressed on a dry-weight basis.

### 2.4. Preparation of Soy Protein Isolate

Soy protein isolate (SPI) was prepared from non-germinated and germinated soybean flours according to Horax et al. [23], with modifications to the defatting, solvent-separation, and drying procedures. The flour was mixed with hexane at a flour-to-hexane ratio of 1:6 (*w*/*v*) and stirred for 6 h at ambient temperature. The mixture was centrifuged at 10,000× *g* for 20 min at 20 °C, and the hexane-containing supernatant was removed. The extraction was repeated once using fresh hexane. The retained flour residue was subsequently air-dried in a fume hood at ambient temperature for 12 h to remove residual hexane.

The resulting flour was dispersed in DI water at a concentration of 10 g/100 mL, and the pH was adjusted to 9.0 using 1 M NaOH. The dispersion was stirred for 2 h and centrifuged at 10,000× *g* for 20 min at 20 °C to collect the supernatant. The remaining residue was re-extracted once under the same conditions, and the two supernatants were combined. The combined supernatant was adjusted to pH 4.5 using 1 M HCl and centrifuged at 10,000× *g* for 20 min to recover the precipitated protein.

The protein precipitate was washed twice with DI water adjusted to pH 4.5 and centrifuged at 10,000× *g* for 20 min after each washing step. The washed SPI was adjusted to pH 7.0 and dried in a hot-air oven (UFE 600, Memmert Company, Memmert, Germany) at 60 °C until its moisture content reached approximately 7%. Moisture content was determined using the AOAC Official Method [22] and expressed on a dry-weight basis. The dried SPI was packed in aluminium foil pouches and stored at 4 °C until enzymatic hydrolysis and subsequent analyses.

### 2.5. Enzymatic Hydrolysis with Alcalase

Enzymatic hydrolysis was performed according to Horax et al. [23], with modifications. Soybean flour and SPI were separately dispersed in deionized water at a sample-to-water ratio of 2:100 (*w*/*v*), corresponding to 2 g of substrate per 100 mL of dispersion. The initial pH was adjusted to 4.0 using 1 N HCl before enzyme addition. The pH was not subsequently monitored or controlled during the 3-h hydrolysis period. This pH was selected based on Horax et al. [23], who investigated Alcalase hydrolysis of soy protein isolate under acidic conditions (pH 3.0–4.0). Alcalase^®^ 2.4 L was added at 1.5% (*v*/*v*), equivalent to 1.5 mL per 100 mL of dispersion or 0.75 mL per gram of substrate. Enzyme dosage was calculated based on total substrate mass rather than protein content.

For the E40 treatments, the dispersions were hydrolyzed in a shaking water bath at 40 °C and 120 rpm for 3 h. For the E60 treatments, hydrolysis was conducted at 60 °C and 120 rpm for the same duration. Following hydrolysis, Alcalase was inactivated by heating the dispersions at 85 °C for 5 min. The hydrolysates were cooled to ambient temperature and dried in a hot-air oven (UFE 600, Memmert Company, Memmert, Germany) at 60 °C until their moisture content reached approximately 7%. The dried hydrolysates were packed in aluminum foil pouches and stored at 4 °C until further analysis.

### 2.6. Determination of Water-Extractable Protein Content

Water-extractable protein content was determined using the Bradford assay according to Bungthong et al. [24], based on the method of Bradford [25], with modifications. Each powdered sample was extracted with DI water at a sample-to-water ratio of 1:20 (*w*/*v*) for 2 h at ambient temperature without heating. The mixture was centrifuged at 5000× *g* for 10 min using a Universal 320/320R centrifuge (Hettich, Boucherville, QC, Canada), and the resulting supernatant was collected for analysis.

An aliquot (0.5 mL) of the supernatant was mixed with 1.0 mL of Bradford reagent and incubated for 5 min at ambient temperature. Absorbance was measured at 595 nm using a DR 2700™ Portable Spectrophotometer (Hach, Loveland, CO, USA). Bovine serum albumin (BSA) was used as the calibration standard. Water-extractable protein content was calculated based on the BSA calibration curve, extraction volume, sample mass, and dilution factor and expressed as milligrams per gram of sample on a dry basis (mg/g db).

Water-extractable protein contents were expressed as bovine serum albumin (BSA) equivalents. As the Bradford assay response depends on protein composition and peptide characteristics, the reported values represent Bradford-reactive protein equivalents rather than absolute protein concentrations.

### 2.7. Determination of Crude Lipid Content

Crude lipid content was determined using the Soxhlet extraction method according to AOAC Official Method 945.16 [26], with modification to the sample mass. Approximately 3 g of each powdered sample was accurately weighed and extracted with petroleum ether in a Soxhlet apparatus for 6 h. Following extraction, the petroleum ether was removed, and the extraction flask containing the lipid residue was dried in a hot-air oven at 105 °C until a constant weight was attained. The flask was cooled before weighing, and the lipid content was determined gravimetrically and expressed as a percentage on a dry basis (% db).

### 2.8. Determination of Total Phenolic Content

Total phenolic content (TPC) was determined according to Ratseewo et al. [27], with modifications. Each powdered sample (1 g) was extracted three times with 10 mL of an acidified methanolic solution consisting of 80% methanol and 1.0 N HCl (85:15, *v*/*v*). Each extraction was performed at a sample-to-solvent ratio of 1:10 (*w*/*v*) by shaking at 180 rpm for 2 h at ambient temperature. The extracts obtained from the three extraction cycles were combined and used for TPC determination.

An aliquot (0.2 mL) of the sample extract was reacted with 0.5 mL of 10% Folin–Ciocalteu reagent and 2.25 mL of 7% sodium carbonate solution. The mixture was incubated for 90 min, and its absorbance was measured at 725 nm (UV-1700, Shimadzu, Tokyo, Japan). Gallic acid was used as the calibration standard. TPC was expressed as milligrams of gallic acid equivalents per 100 g of sample on a dry basis (mg GAE/100 g db).

### 2.9. Determination of Total Flavonoid Content

Total flavonoid content (TFC) was determined using the colorimetric method described by Siriamornpun et al. [28]. The acidified methanolic extract prepared as described in Section 2.8 was used for the analysis. An aliquot of the extract (0.5 mL) was mixed with 2.25 mL of distilled water, followed by 0.15 mL of 5% NaNO_2_ solution. After 6 min, 0.3 mL of 10% AlCl_3_·6H_2_O solution was added. The mixture was allowed to stand for a further 5 min before 1.0 mL of 1 M NaOH was added and mixed by vortexing. Absorbance was measured immediately at 510 nm using a spectrophotometer (UV-1700, Shimadzu, Tokyo, Japan). TFC was calculated using a rutin calibration curve and expressed as milligrams of rutin equivalents per 100 g of sample on a dry-weight basis (mg RE/100 g db).

### 2.10. Determination of DPPH Radical-Scavenging Activity

DPPH radical-scavenging activity was determined according to Ratseewo et al. [27], with modification to the expression of the results. The acidified methanolic extract prepared as described in Section 2.8 was used for the analysis. An aliquot (0.1 mL) of the sample extract was mixed with 3.0 mL of 0.001 M DPPH solution prepared in methanol. The mixture was incubated in the dark at ambient temperature for 30 min, after which the absorbance was measured at 517 nm using a spectrophotometer (UV-1700, Shimadzu, Tokyo, Japan).

Trolox was used to construct the calibration curve. DPPH radical-scavenging activity was calculated from the Trolox calibration curve and expressed as milligrams of Trolox equivalents per gram of sample on a dry basis (mg TE/g db).

### 2.11. Determination of Ferric-Reducing Antioxidant Power

Ferric-reducing antioxidant power (FRAP) was determined according to Ratseewo et al. [27], with modification to the calibration standard and expression of the results. The acidified methanolic extract prepared as described in Section 2.8 was used for the analysis.

The FRAP reagent was freshly prepared by mixing 300 mM acetate buffer (pH 3.6), 10 mM TPTZ prepared in 40 mM HCl, and 20 mM FeCl_3_·6H_2_O at a ratio of 10:1:1 (*v*/*v*/*v*). An aliquot (60 µL) of the sample extract was mixed with 180 µL of freshly prepared FRAP reagent and incubated at 37 °C for 4 min. Absorbance was measured at 593 nm (UV-1700, Shimadzu, Tokyo, Japan). Ferrous sulfate (FeSO_4_) was used to construct the calibration curve, and the FRAP value was expressed as milligrams of FeSO_4_ equivalents per gram of sample on a dry basis (mg FeSO_4_/g db).

### 2.12. Determination of GABA Content

GABA was extracted using a method adapted from Jannoey et al. [29]. Finely ground sample (250 mg) was mixed with 800 µL of 70% methanol, vortexed for 1 min, and left to stand for 30 min. The mixture was centrifuged at 13,000 rpm and 4 °C for 20 min. The supernatant was then filtered through a 0.2 µm membrane filter before LC–MS/MS analysis.

GABA was determined using an LCMS-8030 triple-quadrupole mass spectrometer (Shimadzu, Kyoto, Japan) equipped with an electrospray ionization source, with analytical conditions adapted from Eckstein et al. [30]. Separation was performed on an Inertsil^®^ ODS-3 C18 column (2.1 × 100 mm, 3 µm; GL Sciences, Tokyo, Japan) maintained at 28 °C. The mobile phase consisted of 1% formic acid solution (A) and methanol (B) at a constant ratio of 50:50 (*v*/*v*), delivered at 0.2 mL/min over a 10 min run. The injection volume was 2 µL. Mass spectrometric detection was performed in positive-ion mode using the selected reaction monitoring transition *m*/*z* 104.1 → 87.1. The interface voltage was 4.5 kV; the Q1 pre-bias and collision energy were −18 and −13 V, respectively. The nebulizing and drying gas flow rates were 3 and 15 L/min, respectively, the heating block temperature was 400 °C, and the collision gas pressure was 230 kPa. Instrument control and data processing were performed using LabSolutions software (version 5.53 SP3; Shimadzu).

GABA was quantified using an external calibration curve prepared from a 100 µg/mL GABA stock solution. Seven calibration concentrations ranging from 0.122 to 15.625 µg/mL were used. Linear regression of peak area (y) against GABA concentration (x, µg/mL) yielded the equation y = 194,136.0531x + 21,929.8065, with a coefficient of determination (R^2^) of 0.9995. The estimated limits of detection (LOD) and quantification (LOQ) were 1.190 and 3.606 µg/mL, respectively, and the reported recovery ranged from 95.2% to 114.8%. GABA concentrations were calculated using the calibration equation and expressed as milligrams per gram of sample on a dry-weight basis (mg/g db), accounting for the extraction volume, dilution factor, sample mass, and moisture content.

### 2.13. Amino Acid Analysis

Amino acids were extracted and determined according to Ratseewo et al. [27]. Ground sample (200 mg) was extracted with 10 mL of methanol for 3 min using a sonication probe at 50% amplitude with 50-s on/50-s off cycles (VCX-750 Vibra-Cell^®^, Sonics and Materials Inc., Newtown, CT, USA). The mixture was centrifuged at 19,000× *g* for 10 min, and the remaining pellet was re-extracted under the same conditions. The combined supernatants were evaporated under vacuum at 40 °C, and the residue was re-dissolved in 5 mL of methanol and filtered through a 0.22-µm nylon membrane. Free amino acids were analyzed using a Shimadzu LCMS-8030 triple-quadrupole mass spectrometer equipped with an electrospray ionization source and coupled to a Shimadzu HPLC system (Shimadzu, Kyoto, Japan), following the amino acid analysis procedure described by Chumroenphat et al. [31]. The gradient elution program, injection volume, run time, ionization polarity, and analyte-specific MS/MS acquisition parameters, including the monitored ion transitions and collision energies, were identical to those reported in that study. Chromatographic separation was performed using an InertSustain^®^ C18 column (2.1 mm × 150 mm, 3 µm particle size). The flow rate was 0.2 mL/min, and the autosampler and column oven temperatures were maintained at 4 and 38 °C, respectively. Mobile phase A consisted of water containing 0.1% formic acid (*v*/*v*), and mobile phase B consisted of methanol–water (50:50, *v*/*v*) containing 0.1% formic acid (*v*/*v*). The gradient elution and MS/MS conditions followed the amino acid analysis procedure described by Chumroenphat et al. [31]. Free amino acids were quantified using external calibration curves prepared from the corresponding amino acid standards. The analyte-specific multiple reaction monitoring (MRM) transitions, Q1 and Q3 pre-bias voltages, and collision energies are provided in Supplementary Table S1. Calibration ranges, regression equations, coefficients of determination (R^2^), and reported analytical performance parameters are summarized in Supplementary Table S2. Free amino acid concentrations were calculated from their respective calibration equations and expressed as micrograms per gram of sample on a dry-weight basis (µg/g db), accounting for sample preparation, dilution factors, and moisture content. Three independent processing batches were prepared for each treatment. One sample aliquot from each batch was extracted separately and analyzed by LC–MS/MS. The three batch-level measurements represented independent experimental replicates (n = 3), rather than repeated instrumental injections of the same sample.

### 2.14. Statistical Analysis

The experiment was conducted using a completely randomized design (CRD) comprising 15 treatments, with three independently prepared batches per treatment. Each batch represented one independent experimental replicate and was considered one experimental unit (n = 3 per treatment). The results were expressed as mean ± standard deviation (SD) of the three independent batches. Data from all 15 treatments were analysed together using one-way analysis of variance (ANOVA). Differences among treatment means were determined using Duncan’s multiple range test at a significance level of *p* < 0.05. Different lowercase letters within the same column indicate significant differences among treatments. Statistical analyses were performed using SPSS software version 22 (IBM Corp., Armonk, NY, USA). The analysis was intended to compare individual processing treatments and did not estimate the independent main effects or interactions of germination duration, hydrolysis temperature, and processing route. Residual normality and homogeneity of variances were not formally assessed. With only three independent processing batches per treatment, estimates of within-treatment variability were based on limited data. The results of the one-way ANOVA and Duncan’s multiple range test should therefore be interpreted with caution.

## 3. Results

### 3.1. Effects of Germination and Enzymatic Hydrolysis on Water-Extractable Protein and Lipid Contents

Water-extractable protein content differed significantly among the 15 processing treatments (Table 1). Water-extractable protein content was higher in the Alcalase-treated soybean flour samples SE40 and SE60 (16.19 and 19.25 mg/g db, respectively) than in untreated soybean flour (S; 13.92 mg/g db). A similar response was observed in germinated soybean flour, with SG24E60 and SG72E60 reaching 20.78 and 20.56 mg/g db, respectively, compared with 16.08 mg/g db in SG24 and 15.03 mg/g db in SG72. Germination induces extensive metabolic changes in seeds, including the mobilisation and modification of storage proteins to provide nutrients required for seedling development [5]. Moreover, proteolytic processes during germination can modify protein composition and properties, thereby affecting protein extractability [5,6]. In the present study, SG24E60 and SG72E60 showed higher Bradford-measured water-extractable protein values (20.78 and 20.56 mg/g db, respectively) than their corresponding non-hydrolyzed flour samples, SG24 and SG72 (16.08 and 15.03 mg/g db, respectively). The higher water-extractable protein contents observed in several Alcalase-treated samples may be associated with changes in protein structure and extractability during enzymatic processing. Previous studies have reported the formation of lower-molecular-weight peptides following Alcalase hydrolysis of soybean proteins [12]. However, the present measurements reflect differences in water-extractable protein content rather than the extent of protein hydrolysis. Previous work has reported changes in protein extraction yield and solubility following enzyme-assisted processing [31]. These findings provide possible explanations for treatment-related differences in the present study; however, the Bradford measurements do not establish differences in the extent of proteolysis. SE60, SG24E60, and SG72E60 showed higher Bradford-measured water-extractable protein values than SE40, SG24E40, and SG72E40, respectively. These comparisons describe differences in the measured assay values and do not establish a greater degree of hydrolysis at 60 °C.

The SPI-derived hydrolysates contained 18.97–23.04 mg/g db water-extractable protein, with the highest content observed in SG72IE60 (23.04 mg/g db), followed by SG24IE60 (22.04 mg/g db). These results demonstrate treatment-related differences in the water-extractable protein fraction among samples obtained through different sequences of germination, SPI preparation, and enzymatic hydrolysis. Protein fractionation and enzyme-assisted extraction can modify the physicochemical characteristics and aqueous extractability of legume proteins, resulting in protein fractions with distinct solubility properties [32]. Thus, the relatively high values observed in the SPI-derived hydrolysates may be associated with differences in sample composition resulting from SPI preparation followed by Alcalase hydrolysis. In contrast, lipid content decreased with germination, from 18.35% db in untreated soybean flour to 16.23 and 15.47% db after 24 and 72 h of germination, respectively. This reduction is consistent with the mobilisation of seed storage reserves during germination, when lipids and other macromolecules are metabolically reorganised to support seedling development [5,6]. Labat et al. [6] similarly demonstrated substantial remodeling of lipid reserves and storage proteins during seed germination. The SPI-derived hydrolysates contained considerably lower lipid levels (3.44–3.93% db), which corresponded to the defatting step included in the SPI preparation procedure. Overall, germination was associated with moderate lipid depletion, whereas the SPI preparation route produced the greatest reduction in lipid content.

The water-extractable protein values should be interpreted with caution because the Bradford assay was calibrated using BSA and may respond differently to intact soybean proteins and peptides generated during Alcalase hydrolysis. Differences among treatments may therefore reflect both changes in the water-extractable protein fraction and variations in assay response. Consequently, these values should not be considered direct measures of total protein recovery or the degree of hydrolysis.

### 3.2. Total Phenolic and Flavonoid Contents and Antioxidant Activities

Phenolic and flavonoid contents and antioxidant capacity differed markedly among the soybean flour treatments (Table 2). Untreated soybean flour (S) contained 25.04 mg GAE/100 g db of TPC and 15.21 mg RE/100 g db of TFC. Germination substantially increased these values, reaching 45.35 mg GAE/100 g db and 30.34 mg RE/100 g db after 24 h (SG24), and 65.47 mg GAE/100 g db and 45.31 mg RE/100 g db after 72 h (SG72), respectively. The highest values were observed when 72 h germination was followed by Alcalase hydrolysis at 60 °C (SG72E60), with TPC and TFC reaching 75.03 mg GAE/100 g db and 55.74 mg RE/100 g db, respectively. The Folin–Ciocalteu values represent apparent total phenolic content because non-phenolic reducing constituents may also contribute to the assay response. Therefore, higher values in the processed samples cannot be attributed exclusively to increased phenolic concentrations, and the contributions of phenolic and non-phenolic constituents were not separately determined. Germination induces metabolic and structural changes in seeds that can modify the synthesis, release, and extractability of phenolic compounds. In soybean, Huang et al. [7] reported increased phenolic compounds, isoflavones, and antioxidant capacity during germination, with substantial changes occurring within three days. More recent evidence also indicates that germination can activate secondary metabolic pathways involved in phenolic biosynthesis and alter the distribution and extractability of free and bound phenolic fractions [33,34]. Thus, the progressive increases in TPC and TFC observed between 24 and 72 h of germination are consistent with the metabolic and compositional changes associated with seed germination.

Alcalase-treated samples generally exhibited higher measurable TPC and TFC than the corresponding non-hydrolysed flour samples. In ungerminated soybean flour, TPC increased from 25.04 mg GAE/100 g db in S to 35.12 and 40.21 mg GAE/100 g db in SE40 and SE60, respectively. A similar pattern was observed in the germinated samples, with hydrolysis at 60 °C consistently producing higher TPC and TFC values than the corresponding treatment at 40 °C. Enzymatic hydrolysis can modify protein structure and protein–phenolic interactions, potentially altering the accessibility and extractability of phenolic constituents within the food matrix. Processing-induced changes in the association between phenolic compounds and macromolecular components have been recognised as important determinants of measurable phenolic content and antioxidant properties [34]. In addition, Alcalase hydrolysis of soybean proteins generates low-molecular-weight peptide fractions with radical-scavenging and reducing properties, which may further contribute to the antioxidant capacity of the resulting hydrolysates [35]. Similarly, Qi et al. [17] reported antioxidant activity in protein hydrolysates prepared from germinated soybeans, with differences among the resulting peptide fractions. The changes in DPPH and FRAP activities generally paralleled those observed for TPC and TFC. DPPH activity increased from 15.22 mg TE/g db in S to 55.73 mg TE/g db in SG72E60, while FRAP increased from 15.14 to 55.91 mg FeSO_4_/g db. SG72E60 consequently exhibited the highest values for all four indices among the flour-based treatments. Antioxidant capacity was also evaluated in defatted soybean flour extracts after 72 h of germination followed by Alcalase hydrolysis [18]. The simultaneous increases in phenolic and flavonoid contents and antioxidant capacity are consistent with previous observations in germinated soybean, in which increases in bioactive compounds were accompanied by enhanced antioxidant activity [7]. Germination time has also been shown to affect the antioxidant capacity of soybean sprouts, although the response varies among antioxidant assays [36]. Recent studies further indicate that germination-induced changes in phenolic and flavonoid metabolism can contribute to the antioxidant properties of germinated plant materials [33,34]. In addition to these phytochemical components, antioxidant peptides generated through Alcalase hydrolysis may contribute to the overall antioxidant response, as soybean protein hydrolysates and their low-molecular-weight fractions have demonstrated appreciable radical-scavenging and reducing activities [35]. SG72E60 showed the highest antioxidant-related indices among the flour-based treatments, although the present design does not permit complete separation of the contributions of individual processing steps.

The SPI-derived hydrolysates exhibited lower TPC, TFC, DPPH, and FRAP values than the corresponding flour-based treatments, although these indices generally increased with germination and hydrolysis temperature within the SPI route. The measured TPC was 29.16 mg GAE/100 g db in SG72IE60 and 18.62 mg GAE/100 g db in SIE40. The corresponding TFC values were 17.35 and 9.84 mg RE/100 g db, respectively. The lower phytochemical contents of the SPI-derived samples may be associated with the partitioning of phenolic compounds during protein fractionation, as phenolics can occur in free and matrix-associated forms whose recovery is strongly affected by extraction and processing conditions [34]. Nevertheless, higher values were generally observed in SPI-derived treatments involving longer germination and hydrolysis at 60 °C, indicating treatment-related differences in the measurable phenolic components and antioxidant properties of the protein-enriched fractions.

### 3.3. GABA Accumulation

**Figure 2:**
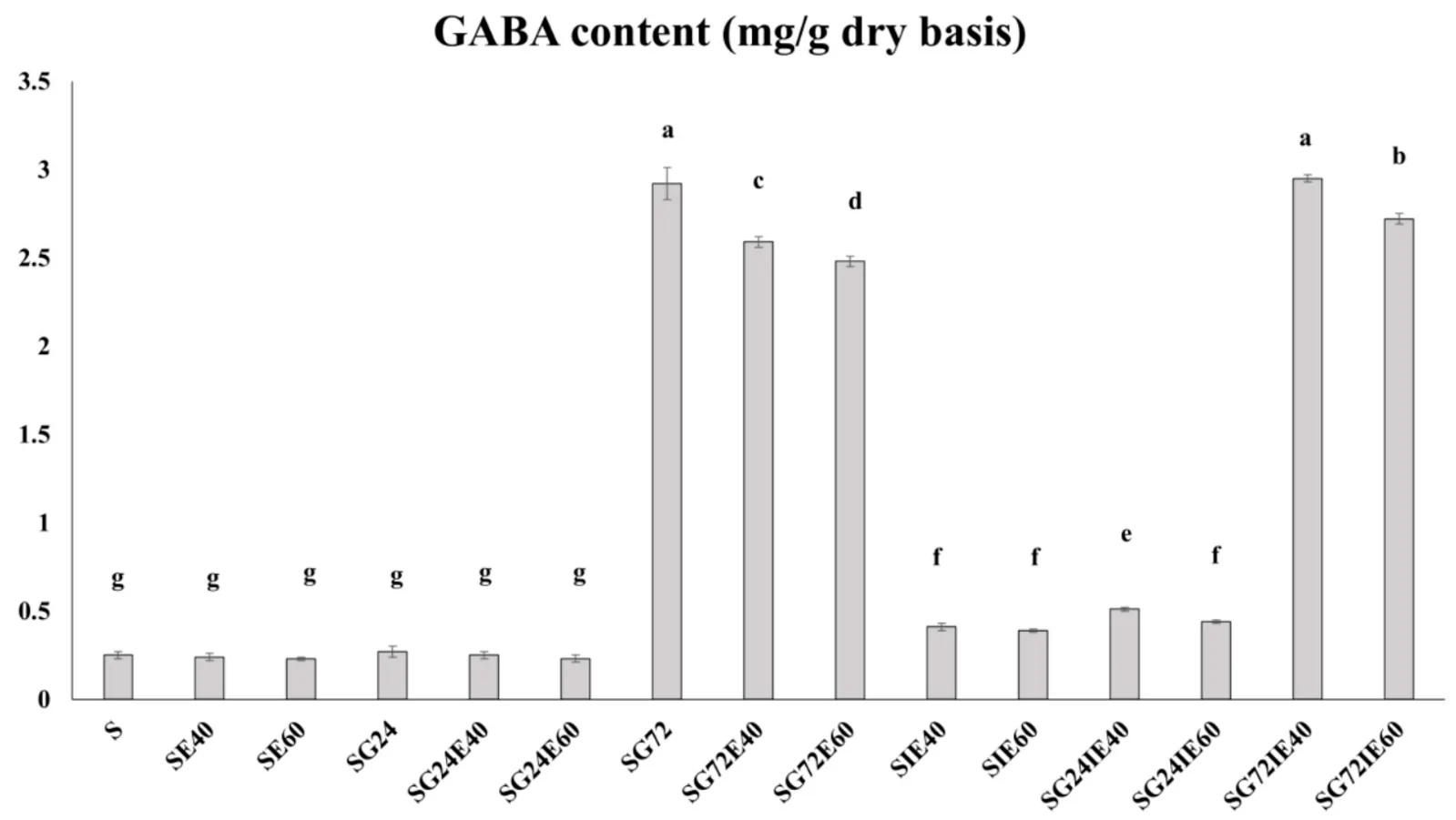
GABA contents (mg/g db) of soybean flour and soy protein isolate samples subjected to germination and enzymatic hydrolysis. Sample codes: S, soybean; G24 and G72, germination for 24 and 72 h, respectively; I, soy protein isolate (SPI) preparation; E40 and E60, Alcalase hydrolysis at 40 and 60 °C, respectively. The position of I before E indicates that SPI was prepared before enzymatic hydrolysis. Values are presented as mean ± SD. Bars with different lowercase letters differ significantly (*p* < 0.05).

GABA accumulation was strongly influenced by germination duration (Figure 2). The untreated soybean flour (S) contained 0.25 mg/g db GABA, while comparable levels were observed after direct Alcalase hydrolysis (0.23–0.24 mg/g) and after 24 h germination, either alone or in combination with hydrolysis (0.23–0.27 mg/g). GABA has also been reported in soybeans by Zazzeroni et al. [37], at a concentration of 211 µg/g. In contrast, extending germination to 72 h markedly increased GABA content to 2.92 mg/g db in SG72, representing an approximately 11.7-fold increase relative to the untreated sample. GABA accumulation during germination is closely associated with the GABA shunt, in which glutamate is converted to GABA through the action of glutamate decarboxylase (GAD). Recent studies have demonstrated substantial increases in GABA during legume germination, accompanied by changes in GAD activity and glutamate metabolism [4,10,38]. In germinated soybean specifically, Xu et al. (2026) [4] further demonstrated that regulation of GAD and GABA transaminase plays an important role in determining GABA accumulation. These findings support the pronounced increase observed after 72 h of germination in the present study.

Following 72 h germination, Alcalase hydrolysis resulted in GABA contents of 2.59 and 2.48 mg/g in SG72E40 and SG72E60, respectively, compared with 2.92 mg/g in SG72. Thus, unlike its effect on water-extractable protein, Alcalase hydrolysis did not further increase GABA content. This difference is consistent with the distinct biochemical processes involved, as Alcalase catalyses the hydrolysis of peptide bonds, whereas GABA accumulation during germination is primarily associated with glutamate metabolism and GAD activity [4]. A similar response was observed in the SPI-derived samples, with GABA contents of 2.95 and 2.72 mg/g in SG72IE40 and SG72IE60, respectively. Overall, the pronounced increase after 72 h indicates that germination duration was the major processing factor associated with GABA enrichment in the present soybean samples, consistent with recent evidence showing time- and condition-dependent GABA accumulation during legume germination [4,10].

### 3.4. Free Essential Amino Acid Profile

The free essential amino acid profile differed among soybean samples subjected to germination and enzymatic hydrolysis (Table 3). Untreated soybean flour (S) contained 46.09 µg/g db of summed quantified amino acids, whereas concentrations of 613.62 and 1027.11 µg/g db were observed in SE40 and SE60, respectively. When germination was followed by enzymatic hydrolysis at 60 °C, the summed concentrations reached 1032.77 µg/g db in SG24E60 and 939.52 µg/g db in SG72E60, corresponding to approximately 22.4 and 20.4 times the concentration in untreated soybean flour, respectively. Although the highest numerical value was observed in SG24E60, it was not significantly different from that in SE60 (*p* > 0.05). Thus, a consistently higher concentration was not observed with prolonged germination before hydrolysis. Germination involves extensive mobilization of seed storage proteins and activation of endogenous proteolytic processes to provide amino acids required for seedling growth, thereby modifying the free amino acid pool of germinated seeds [5,6]. Changes in free amino acid profiles following germination and enzymatic processing have also been reported in legume hydrolysates [38]. Although previous soybean studies have described protein degradation and low-molecular-weight peptide formation [12,13,14], the present study measured free amino acid concentrations rather than peptide size distributions or the degree of hydrolysis.

Among the individual amino acids listed in Table 3, leucine (565.00 µg/g db) predominated in SG72E60, followed by phenylalanine (85.34 µg/g db), arginine (80.92 µg/g db), tryptophan (77.92 µg/g db), and isoleucine (60.45 µg/g db). The highest leucine concentration was observed in SG72E60, although this sample did not have the highest summed concentration, indicating that the processing treatments were associated with different patterns of change among individual amino acids. These differences indicate treatment-related variation in the distribution of quantified free amino acids. Enzyme specificity and substrate characteristics are possible explanations discussed in previous studies [12,14,39], but their contributions were not directly evaluated here. Processing-dependent changes in amino acid profiles have also been reported in shallots subjected to different drying methods [39].

Within the SPI route, the highest summed quantified amino acid concentration was observed in SIE40 (772.48 µg/g db), with leucine (380.30 µg/g db), phenylalanine (210.54 µg/g db), and tryptophan (96.68 µg/g db) as the predominant amino acids. This concentration was lower than the highest value observed in the flour-based route, SG24E60 (1032.77 µg/g db). However, differences between the two routes depended on the treatment: SIE40 contained a higher summed concentration than SE40, whereas lower concentrations were observed in SIE60, SG24IE40, and SG24IE60 than in their corresponding flour-based samples. Conversely, SG72IE40 contained a slightly higher numerical concentration than SG72E40, although these samples were not significantly different (*p* > 0.05). Differences between corresponding flour- and SPI-derived samples represent the outcomes of the complete processing routes. They do not establish differences in substrate accessibility or the degree of hydrolysis. Overall, distinct free amino acid profiles were observed between the flour- and SPI-based processing routes. Under the conditions investigated, the highest numerical summed concentration was observed in SG24E60, while the highest leucine concentration was observed in SG72E60, demonstrating that the treatment associated with the highest total did not necessarily yield the highest concentration of every individual amino acid.

### 3.5. Free Non-Essential Amino Acid Profile

The free non-essential amino acid profile differed among the 15 processing treatments (Table 4). Untreated soybean flour (S) contained 4.46 µg/g db of summed quantified non-essential amino acids, whereas concentrations of 118.88 and 344.78 µg/g db were observed following direct enzymatic hydrolysis in SE40 and SE60, respectively. Germination alone also altered the free amino acid pool, with summed concentrations reaching 33.76 and 76.14 µg/g db after 24 and 72 h, respectively. These changes are consistent with the mobilisation of seed storage proteins during germination, during which endogenous proteolytic activity contributes to the release and redistribution of amino acids required for seedling metabolism [5,6]. A similar increase in free non-essential amino acids following germination was reported for pea flour by Yang et al. [21]. Among the flour-based samples subjected to germination followed by Alcalase hydrolysis, the highest summed concentration was observed in SG24E60 (224.50 µg/g db), followed by SG72E60 (189.81 µg/g db). Both values remained below that of SE60, indicating that germination before hydrolysis did not consistently result in a higher summed concentration. Tyrosine (122.43 µg/g db) and glutamic acid (39.59 µg/g db) were the predominant quantified non-essential amino acids in SG24E60, whereas SG72E60 was characterised mainly by tyrosine (99.72 µg/g db) and glutamic acid (71.40 µg/g db). Soybean storage proteins, particularly glycinin and β-conglycinin, contain substantial proportions of glutamic acid/glutamine residues. However, their presence in storage proteins does not directly establish the extent to which free amino acids are released during hydrolysis. Previous studies have reported structural changes in soybean proteins following Alcalase treatment [12,13,14]. In the present study, the observed differences were limited to the quantified free non-essential amino acid profiles and do not establish differences in protein degradation or peptide size. The present results therefore indicate that distinct non-essential amino acid profiles were associated with different combinations of germination and enzymatic hydrolysis.

Hydrolysis temperature was also associated with differences in the free non-essential amino acid profile. In ungerminated soybean flour, the summed concentration increased from 118.88 µg/g db at 40 °C to 344.78 µg/g db at 60 °C. Corresponding increases were observed from SG24E40 to SG24E60 (150.84 to 224.50 µg/g db) and from SG72E40 to SG72E60 (39.16 to 189.81 µg/g db). Within the SPI-derived samples, SIE60 contained 142.60 µg/g db compared with 109.35 µg/g db in SIE40, while concentrations increased from 38.66 to 157.37 µg/g db between SG24IE40 and SG24IE60 and from 35.33 to 79.24 µg/g db between SG72IE40 and SG72IE60. Thus, higher summed concentrations were observed at 60 °C than at 40 °C in all corresponding treatment pairs. In each corresponding treatment pair, the sample treated at 60 °C showed a higher summed quantified free non-essential amino acid concentration than the sample treated at 40 °C. This pattern describes the measured concentrations and does not demonstrate more extensive proteolysis at 60 °C. Within the SPI route, the highest summed concentration was observed in SG24IE60 (157.37 µg/g db), with tyrosine (88.43 µg/g db) and glutamic acid (27.78 µg/g db) as the predominant quantified non-essential amino acids. Glutamic and aspartic acids may contribute to savoury and umami taste, while the overall balance of free amino acids and peptides can influence the sensory properties of protein hydrolysates [14,40]. However, sensory characteristics were not evaluated in the present study, and these compositional results alone do not establish differences in taste. SG24IE60 contained a lower summed concentration than SG24E60 (224.50 µg/g db), and lower concentrations were likewise observed in each SPI-derived sample than in its corresponding flour-based sample. Protein fractionation can modify the composition and physicochemical state of soybean proteins and consequently influence their subsequent behaviour during enzymatic treatment [19].

Overall, the results from Table 3 and Table 4 indicate that the treatments associated with the highest summed concentrations differed between the essential and non-essential amino acid profiles. The highest numerical sum of quantified essential amino acids was observed in SG24E60 (1032.77 µg/g db), although it was not significantly different from SE60 (1027.11 µg/g db), whereas the highest sum of quantified non-essential amino acids was observed in SE60 (344.78 µg/g db). In contrast, SG72E60 showed the highest TPC, TFC, DPPH, and FRAP values among the flour-based samples. These findings indicate that the treatment associated with the highest antioxidant-related values did not also yield the highest summed free amino acid concentrations. The differences among these responses do not establish a direct causal relationship between free amino acid concentrations and antioxidant-related characteristics. Collectively, the results demonstrate that processing conditions were associated with distinct compositional and bioactive profiles: direct Alcalase hydrolysis at 60 °C yielded the highest summed non-essential amino acid concentration, 24 h germination followed by hydrolysis at 60 °C yielded the highest numerical summed essential amino acid concentration, and 72 h germination followed by hydrolysis at 60 °C yielded the highest antioxidant-related values among the flour-based samples.

## 4. Conclusions

This study compared the compositional and antioxidant-related characteristics of soybean samples obtained from 15 processing treatments involving germination, direct Alcalase hydrolysis of soybean flour, and SPI preparation followed by Alcalase hydrolysis. Treatment-related differences were observed in water-extractable protein, lipid content, GABA, phenolic and flavonoid contents, antioxidant capacity, and free amino acid profiles. The highest numerical sum of quantified free essential amino acids was observed in SG24E60, although it was not significantly different from SE60, whereas the highest sum of quantified free non-essential amino acids was observed in SE60. SG72E60 showed the highest TPC, TFC, DPPH, and FRAP values among the flour-based samples. High GABA concentrations were observed in treatments involving 72 h germination. Hydrolysis at 60 °C yielded higher summed non-essential amino acid concentrations than the corresponding 40 °C treatments in all processing routes, whereas the trend for essential amino acids varied, with a lower summed concentration in SIE60 than in SIE40. The SPI-derived hydrolysates generally showed higher water-extractable protein contents but lower phytochemical and antioxidant-related indices than the corresponding flour-based samples. Differences in summed essential amino acid concentrations between the two routes depended on the treatment, while summed non-essential amino acid concentrations were consistently lower in the SPI-derived samples. Overall, the processing conditions associated with the highest values differed among the measured characteristics, indicating that no single treatment maximised all responses. These findings provide comparative information for selecting processing conditions according to the desired compositional and bioactive characteristics in the further development of soybean-derived ingredients.

## Figures and Tables

**Table 1:** Water-extractable protein and lipid contents of soybean flour and soy protein isolate-derived hydrolysates obtained through germination and Alcalase hydrolysis treatments.

Samples	Water-Extractable Protein Content (mg/g db)	Lipid Content (% db)
S	13.92 ± 0.12 ^i^	18.35 ± 0.22 ^a^
SE40	16.19 ± 0.11 ^g^	18.52 ± 0.25 ^a^
SE60	19.25 ± 0.56 ^ef^	18.31 ± 0.28 ^a^
SG24	16.08 ± 0.84 ^g^	16.23 ± 0.35 ^b^
SG24E40	16.03 ± 0.37 ^g^	16.11 ± 0.31 ^b^
SG24E60	20.78 ± 0.61 ^c^	16.24 ± 0.29 ^b^
SG72	15.03 ± 0.82 ^h^	15.47 ± 0.33 ^c^
SG72E40	15.18 ± 0.46 ^h^	15.34 ± 0.27 ^c^
SG72E60	20.56 ± 0.78 ^cd^	15.11 ± 0.25 ^c^
SIE40	18.97 ± 0.32 ^f^	3.93 ± 0.08 ^d^
SIE60	19.02 ± 0.32 ^f^	3.88 ± 0.08 ^d^
SG24IE40	19.91 ± 0.28 ^de^	3.72 ± 0.07 ^de^
SG24IE60	22.04 ± 0.36 ^b^	3.69 ± 0.07 ^de^
SG72IE40	18.99 ± 0.28 ^f^	3.55 ± 0.07 ^de^
SG72IE60	23.04 ± 0.36 ^a^	3.44 ± 0.12 ^e^

Values are presented as mean ± SD. Different lowercase letters within the same column indicate significant differences (*p* < 0.05). Sample codes: S, soybean; G24 and G72, germination for 24 and 72 h, respectively; I, soy protein isolate (SPI) preparation; E40 and E60, Alcalase hydrolysis at 40 and 60 °C, respectively. The position of I before E indicates that SPI was prepared before enzymatic hydrolysis.

**Table 2:** Total phenolic and flavonoid contents and antioxidant activities (DPPH and FRAP) of soybean flour and soy protein isolate samples subjected to germination and enzymatic hydrolysis.

Samples	TPC (mg GAE/100 g db)	TFC (mg RE/100 g db)	DPPH (mg TE/g db)	FRAP (mg FeSO_4_/g db)
S	25.04 ± 1.02 ^jk^	15.21 ± 0.70 ^j^	15.22 ± 0.80 ^lm^	15.14 ± 0.90 ^lm^
SE40	35.12 ± 1.45 ^h^	20.64 ± 0.95 ^h^	20.39 ± 1.05 ^ij^	20.69 ± 1.12 ^ij^
SE60	40.21 ± 1.45 ^g^	25.68 ± 1.10 ^g^	25.77 ± 1.22 ^g^	25.25 ± 1.35 ^g^
SG24	45.35 ± 1.85 ^f^	30.34 ± 1.25 ^f^	30.91 ± 1.38 ^f^	30.44 ± 1.58 ^f^
SG24E40	50.51 ± 1.92 ^e^	35.11 ± 1.38 ^e^	35.27 ± 1.55 ^e^	35.71 ± 1.75 ^e^
SG24E60	55.63 ± 2.05 ^d^	40.38 ± 1.52 ^d^	40.94 ± 1.68 ^d^	40.22 ± 1.92 ^d^
SG72	65.47 ± 2.18 ^c^	45.31 ± 1.63 ^c^	45.80 ± 1.82 ^c^	45.68 ± 2.05 ^c^
SG72E40	70.24 ± 2.26 ^b^	50.47 ± 1.78 ^b^	50.11 ± 1.95 ^b^	50.19 ± 2.20 ^b^
SG72E60	75.03 ± 2.32 ^a^	55.74 ± 1.90 ^a^	55.73 ± 2.10 ^a^	55.91 ± 2.35 ^a^
SIE40	18.62 ± 0.74 ^m^	9.84 ± 0.39 ^m^	13.74 ± 0.55 ^m^	14.02 ± 0.56 ^m^
SIE60	20.44 ± 0.82 ^lm^	11.26 ± 0.45 ^lm^	15.28 ± 0.61 ^lm^	15.87 ± 0.63 ^lm^
SG24IE40	22.38 ± 0.90 ^kl^	12.68 ± 0.51 ^kl^	16.91 ± 0.68 ^kl^	17.35 ± 0.69 ^kl^
SG24IE60	24.57 ± 0.98 ^jk^	14.11 ± 0.56 ^jk^	18.66 ± 0.75 ^jk^	19.14 ± 0.77 ^jk^
SG72IE40	26.83 ± 1.07 ^ij^	15.92 ± 0.64 ^ij^	21.35 ± 0.85 ^hi^	21.88 ± 0.88 ^hi^
SG72IE60	29.16 ± 1.17 ^i^	17.35 ± 0.69 ^i^	23.14 ± 0.93 ^h^	23.76 ± 0.95 ^gh^

Values are presented as mean ± SD. Different lowercase letters within the same column indicate significant differences (*p* < 0.05). Sample codes: S, soybean; G24 and G72, germination for 24 and 72 h, respectively; I, soy protein isolate (SPI) preparation; E40 and E60, Alcalase hydrolysis at 40 and 60 °C, respectively. The position of I before E indicates that SPI was prepared before enzymatic hydrolysis.

**Table 3:** Free essential amino acid contents of soybean flour and soy protein isolate samples subjected to germination and enzymatic hydrolysis.

Samples	Essential Amino Acids Content (µg/g db)									
	Leucine	Isoleucine	Lysine	Phenylalanine	Threonine	Tryptophan	Valine	Arginine*	Histidine*	Sum of Quantified Amino Acids
S	8.17 ± 1.26 ^n^	2.21 ± 0.06 ^h^	0.43 ± 0.01 ^i^	0.08 ± 0.002 ^i^	Trace	31.87 ± 1.37 ^f^	0.86 ± 0.06 ^k^	2.23 ± 0.09 ^ij^	0.24 ± 0.003 ^h^	46.09 ± 2.15 ^m^
SE40	289.04 ± 6.01 ^g^	31.18 ± 2.57 ^d^	7.23 ± 1.12 ^g^	180.82 ± 1.04 ^d^	6.24 ± 0.14 ^c^	65.84 ± 0.98 ^c^	21.04 ± 0.35 ^d^	4.41 ± 0.09 ^hi^	7.82 ± 0.70 ^e^	613.62 ± 6.83 ^f^
SE60	476.53 ± 6.18 ^c^	31.52 ± 2.45 ^d^	29.06 ± 2.19 ^b^	281.12 ± 3.07 ^a^	10.82 ± 0.14 ^b^	97.01 ± 0.57 ^a^	31.00 ± 1.34 ^b^	60.88 ± 0.63 ^c^	9.17 ± 1.03 ^cde^	1027.11 ± 7.87 ^a^
SG24	28.98 ± 2.14 ^m^	7.36 ± 0.61 ^g^	1.05 ± 0.01 ^i^	19.94 ± 1.66 ^k^	0.51 ± 0.01 ^g^	2.14 ± 0.12 ^j^	4.61 ± 0.38 ^j^	23.18 ± 1.10 ^f^	0.47 ± 0.01 ^h^	88.24 ± 3.01 ^L^
SG24E40	367.28 ± 4.95 ^f^	43.42 ± 1.67 ^b^	18.31 ± 0.40 ^e^	202.78 ± 5.07 ^c^	11.10 ± 1.20 ^b^	22.61 ± 1.67 ^g^	41.51 ± 0.94 ^a^	4.85 ± 0.32 ^h^	10.04 ± 0.31 ^cd^	721.90 ± 12.46 ^d^
SG24E60	548.54 ± 4.25 ^b^	59.45 ± 2.25 ^a^	51.25 ± 1.24 ^a^	199.30 ± 1.46 ^c^	6.27 ± 0.13 ^c^	65.72 ± 1.10 ^c^	19.79 ± 1.23 ^d^	73.93 ± 0.86 ^b^	8.52 ± 0.21 ^de^	1032.77 ± 10.65 ^a^
SG72	48.97 ± 3.66 ^L^	9.05 ± 1.41 ^fg^	4.44 ± 0.84 ^h^	37.90 ± 1.88 ^j^	2.01 ± 0.27 ^f^	9.08 ± 1.45 ^i^	8.80 ± 0.93 ^i^	15.23 ± 1.96 ^g^	1.07 ± 0.02 ^gh^	136.55 ± 5.15 ^k^
SG72E40	143.53 ± 9.47 ^k^	10.67 ± 1.61 ^efg^	14.12 ± 0.07 ^f^	82.59 ± 6.16 ^i^	2.38 ± 0.15 ^ef^	21.35 ± 1.68 ^g^	15.61 ± 1.32 ^e^	1.90 ± 0.58 ^ij^	2.23 ± 0.04 ^fg^	294.38 ± 11.63 ^j^
SG72E60	565 ± 4.31 ^a^	60.45 ± 2.78 ^a^	19.58 ± 1.65 ^e^	85.34 ± 1.46 ^i^	12.95 ± 1.96 ^a^	77.92 ± 3.80 ^b^	11.25 ± 1.13 ^fgh^	80.92 ± 3.49 ^a^	26.11 ± 1.52 ^a^	939.52 ± 10.05 ^b^
SIE40	380.30 ± 6.49 ^e^	32.02 ± 2.78 ^b^	7.00 ± 1.14 ^g^	210.54 ± 2.16 ^b^	10.47 ± 0.44 ^b^	96.68 ± 0.94 ^a^	27.12 ± 1.50 ^c^	0.73 ± 0.02 ^j^	7.62 ± 0.94 ^fe^	772.48 ± 7.75 ^c^
SIE60	410 ± 3.68 ^d^	38.32 ± 2.10 ^c^	15.23 ± 2.11 ^f^	84.60 ± 3.20 ^i^	10.95 ± 0.80 ^b^	45.20 ± 1.50 ^e^	28.40 ± 1.90 ^c^	3.30 ± 1.20 ^hij^	12.80 ± 0.90 ^b^	648.80 ± 7.94 ^e^
SG24IE40	153.40 ± 6.59 ^j^	12.41 ± 3.25 ^ef^	2.23 ± 0.15 ^i^	97.63 ± 0.71 ^g^	3.39 ± 0.11 ^e^	24.03 ± 1.39 ^g^	9.90 ± 0.20 ^hi^	2.00 ± 0.05 ^ij^	3.62 ± 0.07 ^f^	308.61 ± 7.52 ^i^
SG24IE60	233.12 ± 2.57 ^i^	13.43 ± 0.74 ^e^	27.05 ± 1.17 ^c^	142.21 ± 0.34 ^e^	4.53 ± 0.21 ^d^	48.59 ± 0.53 ^d^	11.90 ± 0.17 ^fg^	50.15 ± 1.07 ^d^	3.42 ± 0.16 ^f^	534.40 ± 3.19 ^g^
SG72IE40	152.60 ± 6.62 ^j^	9.60 ± 0.74 ^fg^	2.24 ± 0.19 ^i^	90.91 ± 0.73 ^h^	3.10 ± 0.20 ^ef^	23.80 ± 0.48 ^g^	10.31 ± 0.87 ^ghi^	3.22 ± 0.04 ^hij^	3.14 ± 0.25 ^f^	298.92 ± 6.78 ^ij^
SG72IE60	246.15 ± 3.56 ^h^	43.14 ± 2.69 ^b^	22.54 ± 1.35 ^d^	110 ± 4.11 ^f^	4.80 ± 0.12 ^d^	16.33 ± 1.78 ^h^	12.45 ± 1.18 ^f^	41.63 ± 2.81 ^e^	10.40 ± 2.64 ^c^	507.44 ± 9.15 ^h^

Sample codes: S, soybean; G24 and G72, germination for 24 and 72 h, respectively; I, soy protein isolate (SPI) preparation; E40 and E60, Alcalase hydrolysis at 40 and 60 °C, respectively. The position of I before E indicates that SPI was prepared before enzymatic hydrolysis. Values are presented as mean ± SD. Different lowercase letters within the same column indicate significant differences (*p* < 0.05). Trace, amount below the minimum concentration of the standard used for quantification. Arginine* = essential amino acid for growing infants and children. Histidine* = essential amino acid for children.

**Table 4:** Free non-essential amino acid contents of soybean flour and soy protein isolate samples subjected to germination and enzymatic hydrolysis.

Samples	Non-Essential Amino Acid Content (µg/g db)							
	Aspartic Acid	Serine	Asparagine	Tyrosine	Alanine	Glutamic Acid	Glutamine	Sum of Quantified Amino Acids
S	0.10 ± 0.01 ^e^	0.07 ± 0.002 ^i^	0.46 ± 0.03 ^g^	2.92 ± 0.15 ^ij^	0.32 ± 0.01 ^j^	0.26 ± 0.01 ^k^	0.33 ± 0.02 ^hi^	4.46 ± 0.07 ^k^
SE40	18.25 ± 0.46 ^b^	1.31 ± 0.03 ^g^	1.21 ± 0.14 ^f^	28.24 ± 1.23 ^f^	14.42 ± 0.26 ^f^	50.69 ± 0.19 ^e^	4.76 ± 0.25 ^f^	118.88 ± 1.38 ^g^
SE60	16.39 ± 0.55 ^b^	11.76 ± 0.13 ^b^	16.14 ± 0.45 ^a^	167.89 ± 7.79 ^a^	19.93 ± 0.61 ^c^	86.38 ± 1.07 ^b^	26.29 ± 0.34 ^a^	344.78 ± 7.93 ^a^
SG24	1.14 ± 0.08 ^e^	0.30 ± 0.02 ^hi^	5.12 ± 0.35 ^d^	10.17 ± 2.55 ^g^	3.11 ± 0.12 ^i^	13.91 ± 1.36 ^j^	0.01 ± 0.001 ^i^	33.76 ± 2.91 ^j^
SG24E40	23.95 ± 1.89 ^a^	2.84 ± 0.08 ^e^	Trac	3.80 ± 0.17 ^hij^	18.90 ± 1.61 ^cd^	97.62 ± 2.79 ^a^	3.73 ± 0.02 ^f^	150.84 ± 3.74 ^e^
SG24E60	12.46 ± 0.15 ^c^	9.37 ± 0.26 ^c^	14.16 ± 0.29 ^b^	122.43 ± 1.68 ^b^	3.22 ± 0.08 ^i^	39.59 ± 1.03 ^f^	23.27 ± 1.07 ^b^	224.50 ± 2.28 ^b^
SG72	1.13 ± 0.03 ^e^	0.41 ± 0.02 ^h^	4.53 ± 0.28 ^e^	48.68 ± 3.64 ^e^	5.79 ± 0.67 ^h^	15.18 ± 1.54 ^j^	0.42 ± 0.02 ^ghi^	76.14 ± 4.02 ^i^
SG72E40	0.95 ± 0.06 ^e^	Trace	Trace	0.93 ± 0.07 ^j^	10.76 ± 0.42 ^g^	25.45 ± 2.56 ^h^	1.07 ± 0.06 ^ghi^	39.16 ± 2.68 ^j^
SG72E60	0.45 ± 0.01 ^e^	Trace	Trace	99.72 ± 1.22 ^c^	18.24 ± 1.05 ^de^	71.40 ± 5.47 ^c^	Trac	189.81 ± 1.21 ^c^
SIE40	21.80 ± 0.47 ^a^	2.28 ± 0.06 ^f^	Trace	1.36 ± 0.099 ^ij^	22.92 ± 0.09 ^b^	50.54 ± 0.43 ^e^	10.45 ± 1.18 ^d^	109.35 ± 1.67 ^h^
SIE60	18.40 ± 0.85 ^b^	13.20 ± 0.45 ^a^	Trace	8.30 ± 4.20 ^gh^	26.80 ± 0.90 ^a^	60.50 ± 2.10 ^d^	15.40 ± 1.20 ^c^	142.60 ± 5.07 ^f^
SG24IE40	5.19 ± 0.16 ^d^	0.13 ± 0.002 ^hi^	Trace	Trac	11.88 ± 0.21 ^g^	19.54 ± 1.04 ^i^	1.92 ± 0.03 ^g^	38.66 ± 1.10 ^j^
SG24IE60	4.87 ± 0.01 ^d^	8.20 ± 0.17 ^d^	10.64 ± 0.08 ^c^	88.43 ± 2.53 ^d^	11.11 ± 0.04 ^g^	27.78 ± 0.29 ^gh^	6.34 ± 0.34 ^e^	157.37 ± 2.58 ^d^
SG72IE40	2.08 ± 0.02 ^e^	Trace	Trace	Trace	11.90 ± 0.07 ^g^	19.69 ± 0.73 ^i^	1.66 ± 0.04 ^gh^	35.33 ± 0.73 ^j^
SG72IE60	1.11 ± 0.046 ^e^	Trace	Trace	6.41 ± 0.9 ^ghi^	17.25 ± 1.13 ^e^	29.56 ± 0.58 ^g^	24.91 ± 2.31 ^a^	79.24 ± 7.31 ^i^

Sample codes: S, soybean; G24 and G72, germination for 24 and 72 h, respectively; I, soy protein isolate (SPI) preparation; E40 and E60, Alcalase hydrolysis at 40 and 60 °C, respectively. The position of I before E indicates that SPI was prepared before enzymatic hydrolysis. Values are presented as mean ± SD. Different lowercase letters within the same column indicate significant differences (*p* < 0.05). Trace, amount below the minimum concentration of the standard used for quantification.

## Data Availability

The original contributions presented in this study are included in the article/Supplementary Materials. Further inquiries can be directed to the corresponding author.

## Supplementary Materials

The following supporting information can be downloaded at: https://www.mdpi.com/article/10.3390/foods15213988/s1, Table S1: Multiple Reaction Monitoring (MRM) conditions for amino acids on LC/MS/MS; Table S2: Calibration curves and coefficients of determination (R^2^) for the quantitative analysis of amino acids.
