# Supplementary material for: Effects of Germination and Alcalase Hydrolysis on the Compositional and Bioactive Properties of Soybean Flour and Soy Protein Isolate
Source: Foods. 2026 Nov 6;15(21):3988. doi: 10.3390/foods15213988 (PMC13763538; doi:10.3390/foods15213988)
Supplement: Supplementary file 1 [file foods-15-03988-s001.zip › foods-4645104-supplementary.pdf]

Supplementary data

Table S1 Multiple Reaction Monitoring **(**MRM) conditions for amino acids on LC/MS/MS

No.	Parameter	Precursor ion $[M+H]^+$(m/z)	Product ion (m/z)	Q1 Pre Bias (V)	Collision energy (V)	Q3 Pre Bias (V)
1	Alanine	90.07	44.09	-15.60	-11.20	-16.20
2	Asparagine	132.82	73.97	-12.20	-17.60	-15.60
3	Arginine	175.35	70.03	-30.60	-25.40	-15.60
4	Aspartic acid	134.11	73.97	-21.60	-15.40	-17.40
5	Cysteine	122.15	59.01	-11.40	-24.80	-27.20
6	Glutamine	146.76	84.13	-25.80	-17.40	-18.20
7	Glutamic acid	148.03	84.16	-14.80	-16.60	-10.40
8	Glycine	76.13	30.01	-12.60	-11.20	-11.20
9	Histidine	156.16	110.21	-9.20	-15.60	-11.20
10	Isoleucine	132.10	86.10	-13.80	-12.40	-9.40
11	Leucine	132.11	86.08	-13.80	-12.40	-9.20
12	Lysine	147.08	84.14	-13.20	-19.40	-23.40
13	Methionine	150.20	103.99	-10.80	-12.60	-23.60
14	Phenylalanine	166.31	120.10	-30.40	-12.40	-15.60
15	Proline	115.88	69.88	-11.60	-16.60	-16.40
16	Serine	105.95	59.94	-18.80	-13.60	-14.40
17	Threonine	120.10	73.90	-12.40	-11.60	-16.60
18	Tryptophan	205.05	146.05	-13.80	-20.80	-19.40
19	Tyrosine	181.79	135.90	-12.60	-15.40	-18.20
20	Valine	117.90	72.06	-11.60	-13.80	-16.40

Note: Leucine and isoleucine were distinguished by retention time, with leucine eluting earlier than isoleucine under the chromatographic conditions used, despite sharing the same precursor and product ions.

Table S2. Calibration curves and coefficients of determination (R^2) for the quantitative analysis of amino acids.

Amino acids	Calibration range (μg/mL)	Regression equation	R^2	LOD (μg/mL)	LOQ (μg/mL)	Recovery (%)
Alanine	0.05-6.26	y = 1408972x - 4256	0.9995	0.159	0.484	87.1–132.6
Asparagine	0.05-50	y = 1326234x - 952	0.9952	4.295	13.099	95.2–104.0
Arginine	0.05- 3.13	y = 437380x - 16155	0.9984	0.165	0.544	97.4–154.4
Aspartic acid	0.05-1.56	y = 574160x - 1580	0.9999	0.048	0.150	87.3–114.9
Cysteine	1.56-25.0	y = 257138x - 13444	0.9859	6.891	21.017	79.5–117.8
Glutamine	0.05-6.25	y = 917690x - 12460	0.9984	0.349	1.102	85.4–149.1
Glutamic acid	0.05-6.26	y = 2088133x - 20310	0.9988	0.249	0.792	81.9–119.0
Glycine	0.05-1.56	y = 287043x - 2292	0.9978	0.124	0.379	73.3–103.5
Histidine	1.56-50	y = 2191419x - 2525329	0.9994	3.479	10.610	90.7–149.0
Isoleucine	0.19-12.50	y = 11495626x + 2235573	0.9979	6.258	19.086	92.4–107.4
Leucine	0.04-6.25	y = 13690664x + 578578	0.9999	0.191	0.581	88.7–102.3
Lysine	0.04-12.5	y = 1791509x - 13558	0.9999	0.370	1.178	77.6–104.3
Methionine	0.05-6.26	y = 708728x - 2986.8	0.9997	0.199	0.607	88.8–102.6
Phenylalanine	0.05-50	y = 2239269x + 1276544	0.9999	3.276	10.365	99.8–133.0
Proline	0.05-1.56	y = 5392531x - 27272	0.9996	0.105	0.320	90.4–110.6
Serine	0.05- 3.13	y = 1093065x - 50689	0.9993	0.183	0.565	80.2–162.8
Threonine	0.05- 3.14	y = 583437x - 11823	0.9998	0.098	0.299	89.0–131.4
Tryptophan	0.05-1.56	y = 1523896x - 11739	0.9995	0.041	0.125	80.4–99.3
Tyrosine	0.05- 3.13	y = 438730x - 3568.6	0.9991	0.091	0.291	70.9–124.8
Valine	0.004-0.06	y = 487302494x + 300478	0.9997	0.031	0.096	94.2–136.2
